# In this month’s Bulletin

**DOI:** 10.2471/BLT.20.000820

**Published:** 2020-08-01

In the editorial section, Qais Alemi et al. (510) discuss what’s needed to reduce pandemic risks for refugee populations. Heather Adair-Rohani (511) portrays the work of Kirk Robert Smith in improving air quality. 

In the news section, Gary Humphreys (514–515) reports on how regulatory agencies are adapting to the need for expedited development and distribution vaccines, tests and treatments for COVID-19. Baruch Fischhoff talks to Fiona Fleck (516–517) about behavioural science and explains why governments need to test the messages used in the context of pandemic response. 

## Cambodia 

### Spillover at the human–bat interface 

Julien Cappelle et al. (539–547) document Nipah virus circulation.

## Kenya, Uganda, United Republic of Tanzania

### What’s available to treat common infections?

Rosanna Lyus et al. (530–538) evaluate national registration of essential antimicrobials.

## Namibia

### Tracking outcomes of childbirth

Steffie Heemelaar et al. (548–557) report maternal near-miss surveillance data.

## Regions of Africa, the Americas, the Eastern Mediterranean, South-East Asia, the Western Pacific

### Reasons that malaria tests don’t work as well

Rebecca Thomson et al. (558–568) review the evidence for genetic changes in *P. falciparum.*

## Global 

### SARS-CoV-2 transmission

Shoi Shi et al. (518–529) estimate the impact of travel restrictions.

### Global health and food security 

Anila Jacob et al. (576–578) argue for inclusion of the ecosystem.

### Digital health innovations

Mellick J Chehade et al. (569–575) propose personal health hubs.

Karin R Jongsma and Fleur Jongepier (579–580) explore value-sensitive design.

